# Lymph Node Metastasis Spread Patterns and the Effectiveness of Prophylactic Neck Irradiation in Sinonasal Squamous Cell Carcinoma (SNSCC)

**DOI:** 10.3389/fonc.2022.793351

**Published:** 2022-05-30

**Authors:** Qian Liu, Yuan Qu, Kai Wang, Runye Wu, Ye Zhang, Xiaodong Huang, Jianghu Zhang, Xuesong Chen, Jingbo Wang, Jianping Xiao, Junlin Yi, Guozhen Xu, Jingwei Luo

**Affiliations:** Department of Radiation Oncology, National Cancer Center/National Clinical Research Center for Cancer/Cancer Hospital, Chinese Academy of Medical Sciences and Peking Union Medical College, Beijing, China

**Keywords:** lymph node spread pattern, lymph node metastasis, sinonasal malignancies, elective neck irradiation, node-negative neck

## Abstract

**Objectives:**

To analyze the incidence and spread of lymph node metastasis (LNM) and the effectiveness of prophylactic neck irradiation in patients with SNSCC.

**Methods:**

A total of 255 patients with SNSCC were retrospectively reviewed. The LNM spread pattern was revealed. The clinical parameters related to LNM, and the prognostic value of elective neck irradiation (ENI) were assessed. A 1:1 matching with propensity scores was performed between ENI group and observation (OBS) group.

**Results:**

The initial LNM rate was 20.8%, and the regional recurrence (RR) rate was 7.5%. Lymphatic spreading in SNSCC followed the common trajectories: a. level Ib ➔ level II ➔ level Va/level III/IV lymph nodes (LNs); b. retropharyngeal lymph nodes (RPLNs) ➔ level II LNs. The most frequently involved site was level II LNs (16.1%), followed by level Ib LNs (10.2%), RPLNs (4.7%), level III LNs (3.2%), level Va LNs (1.6%), level IVa LNs (1.4%) and level VIII LNs (0.8%). The median follow-up time was 105 months. The 5-year overall survival (OS) was 55.7% for N0 patients and 38.5% for patients with initial N+ or N- relapse (p = 0.009). After PSM, the 5-year regional recurrence-free survival was 71.6% and 94.7% (p = 0.046) in OBS and ENI group, respectively. The multivariate analysis showed that ENI (p = 0.013) and absence of nasopharynx involvement (p = 0.026) were associated with a significantly lower RR rate.

**Conclusions:**

Patients with LNM had poorer survival than those who never experienced LNM. Lymphatic spread in SNSCC followed predictable patterns. ENI effectively reduced the RR rate in patients at high risk.

## Introduction

Sinonasal malignancies (SNMs) account for 3%~5% of all head and neck cancers (1, 2) and constitute a broad spectrum of histopathologic subtypes, of which squamous cell carcinoma represents 50%~80% (3). However, due to the insidiousness of symptoms in the early stage and primary tumors being located adjacent to critical structures, the management of SNM is challenging, resulting in a 5-year overall survival (OS) rate of approximately 50% (4).

Recently, several studies reported that regional metastasis was a prognostic factor for survival (5–7). The best management remains unclear for patients with node-negative (N0) necks. During the 1980s, many oncology teams opposed prophylactic neck treatment due to the rarity of regional metastases (8, 9). However, from the 1990s to the 2000s, the MD Anderson group (10) and Paulino et al. (11) advocated for elective ipsilateral neck irradiation in all patients with maxillary sinus squamous cell carcinoma because they found that up to 33% of N0 patients would eventually present regional failure during the follow-up after a ‘watch and wait’ strategy. Since then, the debate has continued regarding whether elective neck irradiation (ENI) should be performed for N0 sinonasal cancers. The National Comprehensive Cancer Network (NCCN) guidelines recommend ENI in patients with T3-4 disease based on the rationale that ENI of the N0 neck is warranted if the probability of occult cervical metastasis is greater than 20% (12).

Nevertheless, several questions remain unsolved. First, the incidence of lymph node metastasis (LNM) at SNSCC presentation varies widely from 3% to 20.6%, with differences based on race, histopathology, T stage, involved structure and treatment of the primary tumor (13). Second, the prediction of the likely location of regional metastasis is essential but challenging due to the complex lymphatic network of the nasal cavity, paranasal sinuses and neighboring structures. Also, the sentinel lymph node (SLN) approach is hard to implement in SNM (14). Third, the population at high risk for LNM needs to be identified. Except for advanced tumors (T3-4), some investigators found that T2 tumors had a higher rate than more advanced tumors (6). In addition, previous studies reported the invasion of various structures as a risk factor associated with developing regional metastasis, such as invasion of the oral cavity, nasopharynx, hard palate, and sinonasal cavity osseous confines into adjacent structures like the dura, infratemporal fossa and palate (15, 16). Moreover, most studies have included a higher proportion of patients who received no neck treatment; as such, the safety and effectiveness of ENI has not been able to be directly evaluated.

In light of these controversial issues, we conducted a retrospective study in SNSCC to evaluate the influence of LNM on oncology outcomes and LNM incidence and spread patterns. We also analyzed the effectiveness of prophylactic neck irradiation in preventing neck failure and the risk factors associated with nodal involvement at presentation and after treatment.

## Methods and Materials

### Patients

**Figure 1:**
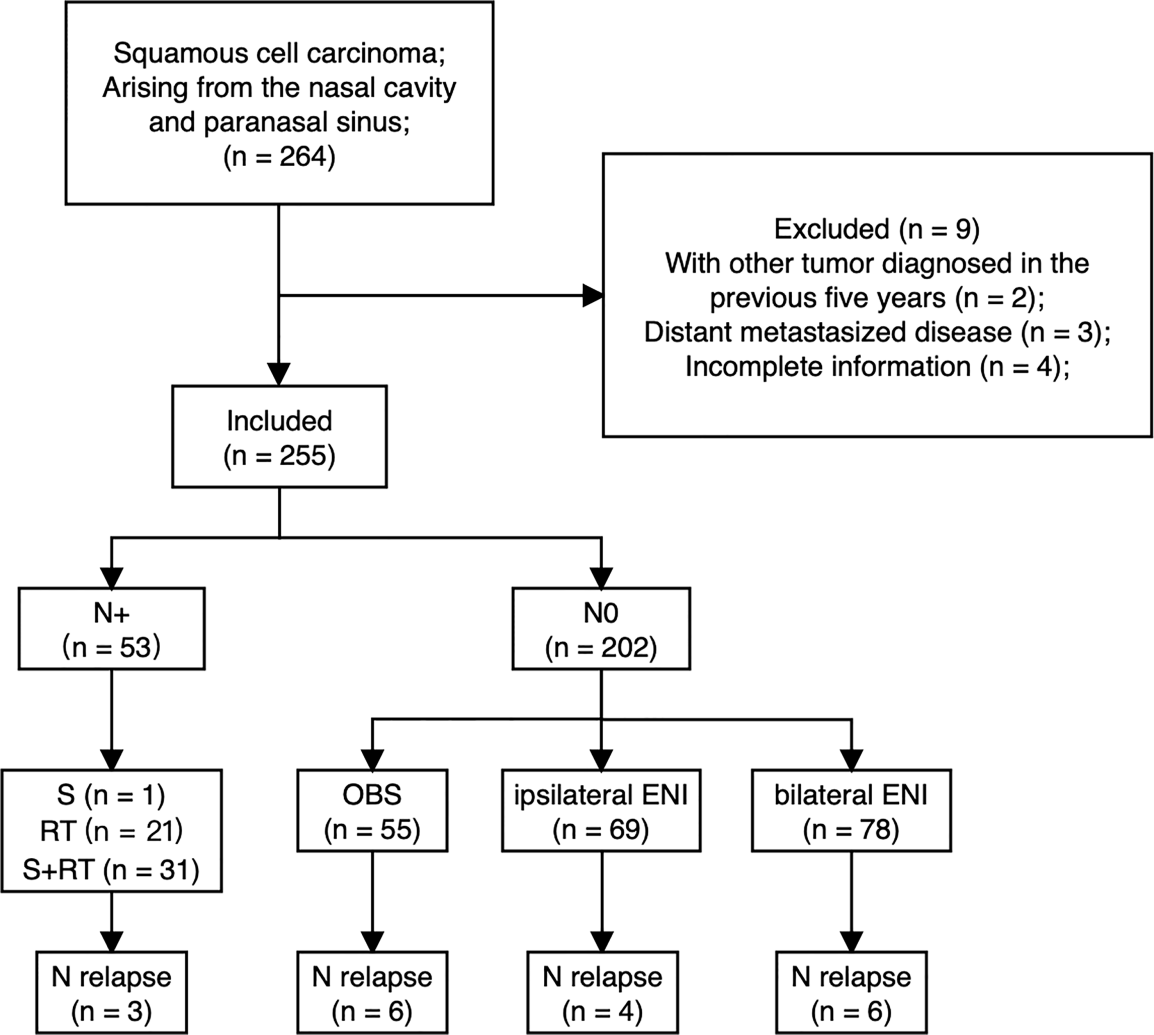
Patient selection and treatment flow chart.

Between Jan 1999 and Dec 2016, consecutive patients with a histopathological diagnosis of squamous cell carcinoma arising from the nasal cavity and paranasal sinus at a single academic tertiary referral center were included. Patients were excluded if they had a new malignant tumor diagnosed in the previous five years, if distant metastases were present at diagnosis, or if clinicopathologic and follow-up information were incomplete. The patient selection and treatment flow chart are depicted in **Figure 1**.

All patients were restaged according to the 8^th^ edition of the AJCC staging system. Clinical LNM was determined by the results of pretreatment imaging examinations (CT/MRI): a minimal axial diameter (MID) of cervical LNs ≥ 10 mm, and a MID of retropharyngeal lymph nodes (RPLNs) ≥ 5 mm; nodal grouping, as defined as three or more contiguous LNs, any one of which had an MID ≥ 8 mm; and the presence of signs of necrosis or extracapsular invasion in any sized LN. The pathologic confirmation of LNM was obtained when it was difficult to certain nodal metastases based on imaging.

### Initial Treatment of Primary Tumors

All patients underwent pretreatment evaluation. After clinical assessment and review, the final treatment modality was decided by the multidisciplinary team.

A preoperative radiotherapy (RT) strategy was preferred if the primary tumor had invaded vital organs, like orbital structures or the brain parenchyma. We routinely assessed the tumor response of patients who received preoperative RT at 50 Gy by CT, MRI and/or endoscopy examination. Nonresponders (<80% reduction of primary lesion) underwent resection of the primary tumor and modified neck dissection 4~6 weeks after receipt of preoperative 50 Gy at 2.0 Gy per fraction. Responders (≥80% reduction of primary lesion) received a boost to PTV up to a total dose of 70 Gy.

Postoperative RT was recommended for patients with selected risk factors, including advanced T stage, perineural/lymphatic/vascular invasion, nonnegative surgical margin, and multiple positive nodes with or without extranodal extension. The prescribed dose was 30 fractions of 60 Gy over six weeks. A higher dose (70 Gy) was recommended for patients with extranodal extension or positive margins.

In patients except for the responders who received preoperative RT, RT was considered a definitive treatment for patients who were unfit for or refused surgery. Typically, the prescribed dose based on primary gross tumor volume (GTVp) was 70 Gy within 6.5~7 weeks.

### Initial Treatment of Neck Lymph Nodes

If the patients had clinically positive lymph nodes at presentation, neck dissection, RT, or a combined treatment regimen was considered. The nodal clinical target volume (CTVnd) encompassed all regions with nodal involvement and extended to the adjacent levels. In addition, bilateral treatment was implemented if a tumor approached or crossed the midline or involved some anatomic regions with crossing lymph node drainage, such as the soft palate, oral cavity, and nasopharynx.

No patients with clinical stage N0 disease received elective nodal dissection, and prophylactic neck irradiation was generally administered to patients with T3-4 SNSCC or any patients with T2 disease with rich lymphatic network structure extension. The preferred prophylactic dose of ENI was 50~60 Gy in 30~33 fractions over 6~7 weeks to high-risk regions.

### Systemic Therapy

Systemic therapy was decided by the multidisciplinary team according to clinicopathologic factors, comorbidity, and patient preference. Induction and adjuvant chemotherapy included the TPF and TP regimens. In concurrent chemoradiotherapy cases, patients received cisplatin weekly or triweekly or docetaxel weekly. Alternatively, patients received nimotuzumab as targeted therapy.

### Definition of Endpoints

OS was defined as the duration from the date of initial diagnosis to death due to any cause or the last follow-up. Local recurrence-free survival (LRFS), regional recurrence-free survival (RRFS) and distant metastasis-free survival (DMFS) were defined as the duration from the date of initial diagnosis to the first failure. Instances of locoregional or distant recurrence were documented by biopsy unless there was clear radiographic evidence of disease. Local treatment failure was defined as recurrence at the site of the initial primary tumor, regional treatment failure was defined as the development of recurrence in head and neck lymph nodes, and distant treatment failure was defined as recurrence in an organ outside of the head or neck.

### Statistical Methods

Normally distributed continuous data are presented as the means with ranges and were compared using the independent samples t-test. Nonnormally distributed continuous data are presented as medians with interquartile ranges (IQRs) and were compared using the Mann-Whitney U test. Categorical data are presented as frequencies with percentages and were compared using the chi-square test with correction for continuity when necessary. Logistic regression was performed to estimate predictors of initial LNM. The OS curve was generated using the Kaplan-Meier method with a log-rank comparison, if needed. The instances of local, regional, and distant treatment failure are depicted in cumulative incidence plots and were compared using the Fine & Gray test. Deaths not related to the event of interest were considered as competing risk events. Multivariate Cox regression analysis was carried out to identify prognostic factors associated with lymph node recurrence for the N0 cases. Logistic regression was performed to estimate predictors of ENI or OBS. Propensity scores were calculated given the covariates of variables estimated from the logistic regression mentioned above using another logistic regression model with a caliper of 0.2; 1:1 matching was performed with the nearest-neighbor algorithm. After matching, normally distributed continuous data were compared using the paired-samples t-test (17). The Wilcoxon signed-rank test was used for nonnormally distributed continuous data; categorical data were compared with McNemar’s test. All analyses were 2-sided and used a significance level of p<0.05. Statistical analyses were performed with SPSS version 26 (IBM Corp) and R version 3.2 (http://www.R-project.org).

## Results

### Patient Characteristics

A total of 255 patients with SNSCC were identified. The detailed characteristics of the patients are shown in **Table 1**.

**Table 1:** Patients’ characteristics.

Variables	Nasal Cavityn = 76 (29.8%)	Maxillary Sinusn = 149 (58.4%)	Ethmoid Sinusn = 30 (11.8%)	Totaln = 255 (100%)
Median age (range)	53 (11~85)	56 (16~83)	49 (14~75)	54 (11~85)
Age				
≤50	36 (47.40%)	48 (32.20%)	16 (53.30%)	100 (39.20%)
>50	40 (52.60%)	101 (67.80%)	14 (46.70%)	155 (60.80%)
Sex				
Female	19 (25.0%)	39 (26.20%)	7 (23.30%)	65 (25.50%)
Male	57 (75.0%)	110 (73.8%)	23 (76.7%)	190 (74.5%)
Year of diagnosis				
1999-2007	31 (40.80%)	51 (34.20%)	16 (53.30%)	98 (38.40%)
2008-2016	45 (59.20%)	98 (65.80%)	14 (46.70%)	157 (61.60%)
AJCC Stage				
I	5 (6.6%)	0 (0.0%)	0 (0.0%)	5 (2.0%)
II	6 (7.9%)	5 (3.4%)	0 (0.0%)	11 (4.3%)
III	19 (25.0%)	31 (20.8%)	2 (6.7%)	52 (20.4%)
IVA	25 (32.9%)	68 (45.6%)	10 (33.3%)	103 (40.4%)
IVB	21 (27.6%)	45 (30.2%)	18 (60.0%)	82 (33.0%)
T stage				
T1	5(6.6%)	0 (0.0%)	0 (0.0%)	5 (2.0%)
T2	9 (11.8%)	6 (4.00%)	0 (0.0%)	15 (5.9%)
T3	20 (26.30%)	34 (22.8%)	2 (6.7%)	56 (22.0%)
T4a	21(27.6%)	67 (45.0%)	10 (33.3%)	98 (38.40%)
T4b	21(27.6%)	42 (28.2%)	18 (60.0%)	81 (31.80%)
N stage				
N0	56 (73.7%)	120 (80.5%)	26 (86.7%)	202 (79.2%)
N1	7 (9.20%)	14 (9.4%)	1(3.30%)	22 (8.6%)
N2	12 (15.8%)	13 (8.7%)	3 (10.0%)	28 (11.0%)
N3	1 (1.3%)	2 (1.3%)	0 (0.0%)	3 (1.2%)
Primary tumor treatment modality				
S+RT	35 (46.10%)	51 (34.20%)	9 (30.00%)	95 (37.30%)
RT+S	16 (21.10%)	56 (37.60%)	9 (30.00%)	81 (31.80%)
RT	19 (25.00%)	36 (24.20%)	11 (36.70%)	66 (25.90%)
S	6 (7.90%)	6 (4.00%)	1 (3.30%)	13 (5.10%)
N+ neck treatment modality				
S	0 (0.0%)	3 (10.3%)	0 (0.0%)	3 (5.7%)
RT	11 (55.0%)	11 (37.9%)	1 (25.0%)	23 (43.4%)
S+RT	9 (45.0%)	15 (51.7%)	3 (75.0%)	27 (50.9%)
N0 neck treatment modality				
ENI	29 (51.8%)	99 (82.5%)	19 (73.1%)	147 (72.8%)
OBS	27 (48.2%)	21 (17.5%)	7 (26.9%)	55 (27.2%)
Systemic therapy				
Chemotherapy	23 (30.30%)	48 (32.20%)	12 (40.00%)	83 (32.50%)
Target therapy	7 (9.20%)	13 (8.70%)	1 (3.30%)	21 (8.20%)
RT technology				
Non-IMRT	32 (45.7%)	54 (37.8%)	13 (44.8%)	99 (40.9%)
IMRT	38 (54.3%)	89 (62.2%)	16 (55.2%)	143 (59.1%)

S, Surgery; RT, Radiotherapy; S+RT, Surgery followed by postoperative radiotherapy; RT+S, preoperative radiotherapy followed by surgery.

Regarding the initial treatment of primary tumors, among 81 patients managed with preoperative RT, the median dose of GTVp was 60 Gy (range: 48~80 Gy). Among 95 patients who received postoperative RT, the median dose of tumor bed volume (GTVtb) was 67 Gy (range: 56~82 Gy).

Regarding the initial neck treatment, for 53 patients with node-positive neck disease, 3 patients underwent neck dissection alone, 23 patients received neck irradiation alone with a median dose of 69.96 Gy (range: 55~80 Gy), and 27 patients received neck dissection combined with irradiation with a median dose was 66 Gy (range: 50~70 Gy). Among 202 patients without clinically metastatic LNs, 147 patients were treated with ENI at a median dose of 60 Gy (range: 50~60 Gy), while 55 patients underwent observation (OBS). Of those who received ENI, 78 (53.1%) received bilateral irradiation, and 69 (46.9%) received ipsilateral irradiation.

Regarding systemic therapy, 83 (32.5%) patients received chemotherapy, and 21 (8.2%) received nimotuzumab targeted therapy. The most common induction or adjuvant chemotherapeutic strategy was the TP regimen (75%, 12/16), while the most common concurrent chemotherapeutic agent was cisplatin (88.9%, 64/72).

### Survival Outcomes

**Figure 2:**
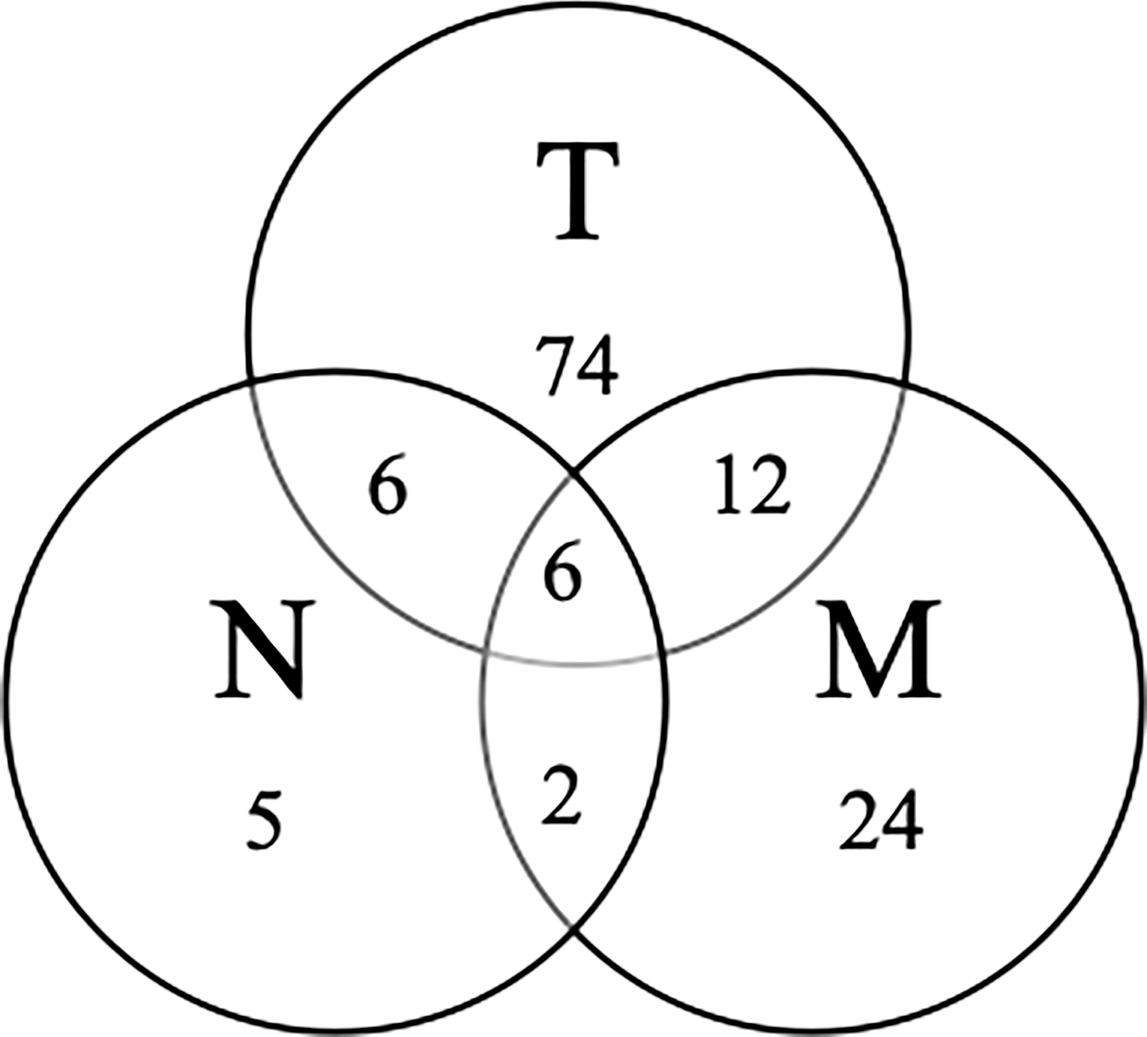
Failure patterns for the 129 patients with SNSCC.

The median follow-up time was 105 months (IQR 65-147 months) in the whole cohort. The 5-year OS and 10-year OS for all patients were 51.3% and 41.3%, respectively. The 5-year OS of the primary tumor site, as ranked from high to low, was as follows: nasal cavity (60.8%), maxillary sinus (51.7%) and ethmoid sinus (27.2%). The treatment failure patterns are summarized in **Figure 2**. At five years, LRFS, RRFS and DMFS were 56.9%, 91.3% and 80.2%, respectively, among all patients.

**Figure 3:**
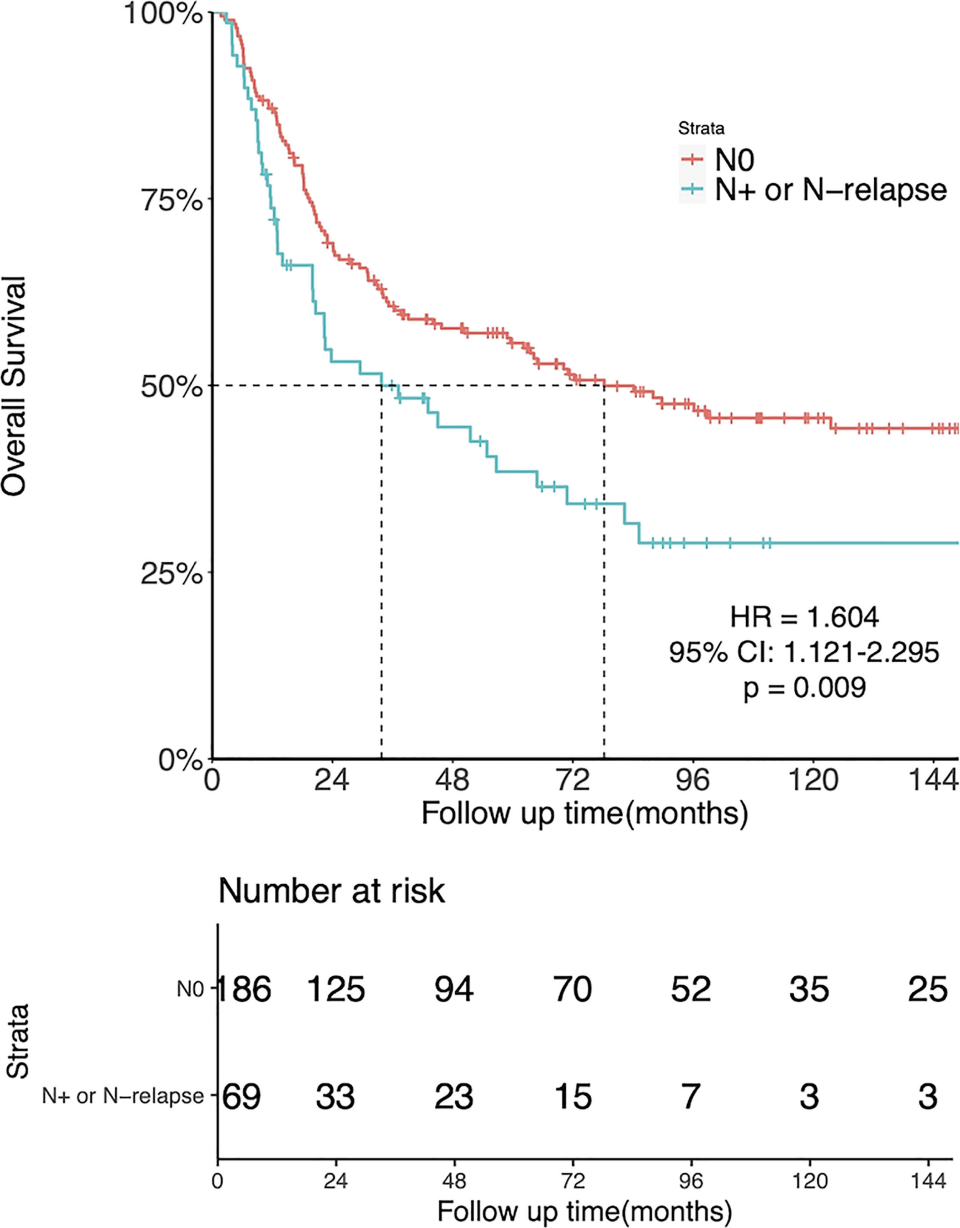
Kaplan-Meier estimates of OS in N0 patients and patients with N+ or N-relapse patients.

There was a significant association between LNM and OS. The 5-year OS was 55.7% for patients with N0 disease and 38.5% for those with initial N+ or N-relapse (HR = 1.604, 95%CI: 1.121-2.295, p = 0.009, **Figure 3**).

### Patients With N0 Disease: ENI vs. OBS

Of all patients, 202 patients with N0 neck at diagnosis, while 53 patients with initial LNM. To evaluate the value of prophylactic neck irradiation in the N0 neck, we compared the outcomes of the ENI (55 patients) and OBS (147 patients) groups. **Table 2** outlines the characteristics of the 202 N0 patients.

**Table 2:** Characteristics of N0 patients undergoing observation (OBS) or elective neck irradiation (ENI) before and after PSM.

	Before PSM		p	After PSM		p
	OBS	ENI		OBS	ENI	
n	55	147		36	36	
Median age (mean (SD))	57.04 (11.64)	53.90 (14.46)	0.151	55.72 (11.70)	50.03 (16.13)	0.091
Sex			0.379			0.792
Male	43 (78.2%)	104 (70.7%)		27 (75.0%)	25 (69.4%)	
Female	12 (21.8%)	43 (29.3%)		9 (25.0%)	11 (30.6%)	
Primary tumor site			<0.001			0.076
Nasal cavity	27 (49.1%)	29 (19.7%)		13 (31.6%)	5 (13.9%)	
Maxillary sinus	21 (38.2%)	99 (67.3%)		16 (44.4%)	24 (66.7%)	
Ethmoid sinus	7 (12.7%)	19 (12.9%)		7 (19.4%)	7 (19.4%)	
T stage			<0.001			0.703
T1	5 (9.1%)	0 (0.0%)		1 (2.8%)	0 (0.0%)	
T2	8 (14.5%)	3 (2.0%)		1 (2.8%)	2 (5.6%)	
T3	14 (25.5%)	33 (22.4%)		8 (22.2%)	9 (25.0%)	
T4	28 (50.9%)	111 (75.5%)		26 (72.2%)	25 (69.4%)	
Treatment modality			<0.001			0.699
S+RT	21 (38.2%)	63 (42.9%)		15 (41.7%)	13 (36.1%)	
RT+S	15 (27.3%)	46 (31.3%)		13 (36.1%)	13 (38.9%)	
RT	7 (12.7%)	38 (25.9%)		7 (19.4%)	9 (25.0%)	
S	12 (21.8%)	0 (0.0%)		1 (2.8%)	0 (0.0%)	
Year of diagnosis			0.001			0.149
1999-2007	34 (61.8%)	50 (34.0%)		25 (69.4%)	18 (50.0%)	
2008-2016	21 (38.2%)	97 (66.0%)		11 (30.6%)	18 (50.0%)	

S, Surgery; RT, Radiotherapy.

**Figure 4:**
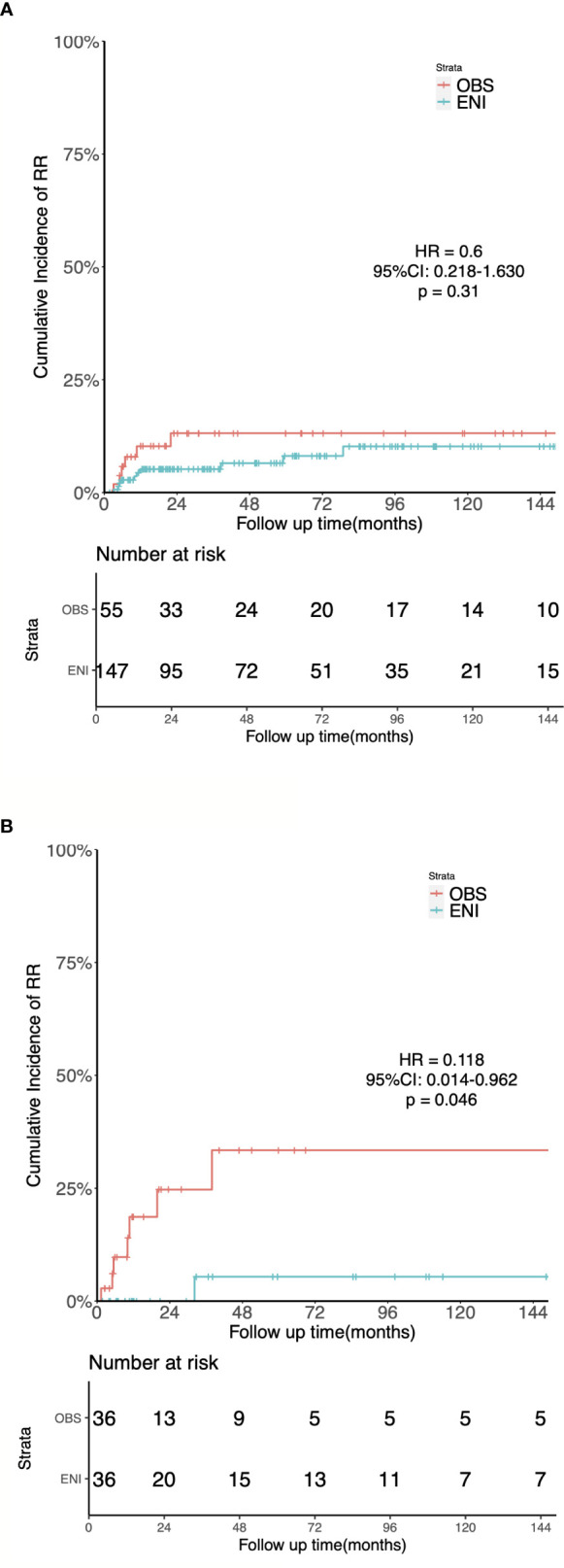
Kaplan-Meier estimates of the cumulative incidence of regional recurrence in the ENI and OBS groups. **(A)** Regional Recurrence in the entire cohort. **(B)** Regional Recurrence in the matched cohort.

For the unmatched group, at a median follow-up time of 111 months (IQR 68-149 months), the 5-year OS was 52.9% and 54% (HR = 1.073; 95%CI: 0.697-1.653; p = 0.748), the 5-year LRFS was 60.1% and 44.6% (HR = 0.661, 95%CI: 0.417-1.05, p = 0.077), the 5-year RRFS was 92.2% and 87.7% (HR = 0.6, 95%CI: 0.218-1.63, p = 0.31, **Figure 4A**), and the 5-year DMFS was 77.9% and 86.5% (HR = 1.9, 95%CI: 0.791-4.58, p = 0.15) in the ENI and OBS group, respectively.

The 1:1 matching for OBS versus ENI resulted in 36 matched pairs, and tests indicated negligible differences across all demographics and clinicopathological variables in the matched cohort.

After PSM, the median follow-up time was 135 months (IQR 42–176 months) for OBS group and 148 months (IQR 65–176 months) for ENI group. Sixteen patients in the OBS group and 15 in the ENI group died. The median OS were 31 and 39 months in the OBS and ENI group, respectively. Additionally, 5-year OS rates was 46.9% in the OBS group and 46.7% (HR = 0.830, 95%CI: 0.449-1.534, p = 0.553), 5-year LRFS was 46.9% and 49.0% (HR = 0.844, 95%CI: 0.449-1.583, p = 0.597), 5-year RRFS was 71.6% and 94.7% (HR = 0.118, 95%CI: 0.014-0.962, p = 0.046, **Figure 4B**), and 5-year DMFS was 76.4% and 75.6% (HR = 1.088, 95%CI: 0.345-3.432, p = 0.886) in the OBS and ENI group, respectively.

In a multivariate Cox regression model (**Table 5**), compared with OBS, ENI resulted in a significantly lower rate of regional failure (HR = 0.169, 95%CI: 0.041-0.690; p = 0.013).

### Incidence and Spread Pattern of Clinically Metastatic LNs

#### LNM Rate and Spread Pattern of LNM at Diagnosis

Of all 255 patients, 53 (20.8%) patients had LNM at diagnosis. Patients with nasal cavity SCC had the highest incidence of LNM (20/76, 26.3%), followed by patients with maxillary sinus SCC (29/149, 19.5%) and those with ethmoid sinus SCC (4/30, 13.3%). The incidence and distribution of LNM based on the primary tumor site are shown in **Table 3**.

**Table 3:** Incidence and distribution of clinically metastatic lymph node at diagnosis.

	Total (n = 255)			Nasal Cavity (n = 76)			Maxillary Sinus (n = 148)			Ethmoid Sinus (n = 30)		
	ipsi-lateral	bi-lateral	contra-lateral	ipsi-lateral	bi-lateral	contra-lateral	ipsi-lateral	bi-lateral	contra-lateral	ipsi-lateral	bi-lateral	contra-lateral
Ib	22 (8.6%)	3 (1.2%)	1 (0.4%)	9 (11.8%)	1 (1.3%)	0 (0.0%)	11 (7.4%)	2 (1.4%)	1 (0.7%)	2 (6.7%)	0 (0.0%)	0 (0.0%)
II	31 (12.2%)	9 (3.5%)	1 (0.4%)	8 (10.5%)	5 (6.6%)	0 (0.0%)	20 (13.5%)	4 (2.7%)	0 (0.0%)	3 (10.0%)	0 (0.0%)	1 (3.3%)
III	6 (2.4%)	2 (0.8%)	0 (0.0%)	2 (2.6%)	1 (1.3%)	0 (0.0%)	3 (2.0%)	1 (0.7%)	0 (0.0%)	1 (3.3%)	0 (0.0%)	0 (0.0%)
IVa	2 (0.8%)	1 (0.4%)	1 (0.4%)	1 (1.3%)	0 (0.0%)	0 (0.0%)	2 (1.4%)	1 (0.7%)	0 (0.0%)	0 (0.0%)	0 (0.0%)	1 (3.3%)
Va	2 (0.8%)	2 (0.8%)	0 (0.0%)	1 (1.3%)	0 (0.0%)	0 (0.0%)	2 (1.4%)	1 (0.7%)	0 (0.0%)	0 (0.0%)	0 (0.0%)	0 (0.0%)
RPN	7 (2.7%)	5 (2.0%)	0 (0.0%)	3 (3.9%)	2 (2.6%)	0 (0.0%)	3 (2.0%)	3 (2.0%)	0 (0.0%)	1 (3.3%)	0 (0.0%)	0 (0.0%)
VIII	2* (0.8%)	0 (0.0%)	0 (0.0%)	0 (0.0%)	0 (0.0%)	0 (0.0%)	1 (0.7%)	0 (0.0%)	0 (0.0%)	1 (3.3%)	0 (0.0%)	0 (0.0%)

*Both patients had pre-auricular lymph node metastasis.

**Figure 5:**
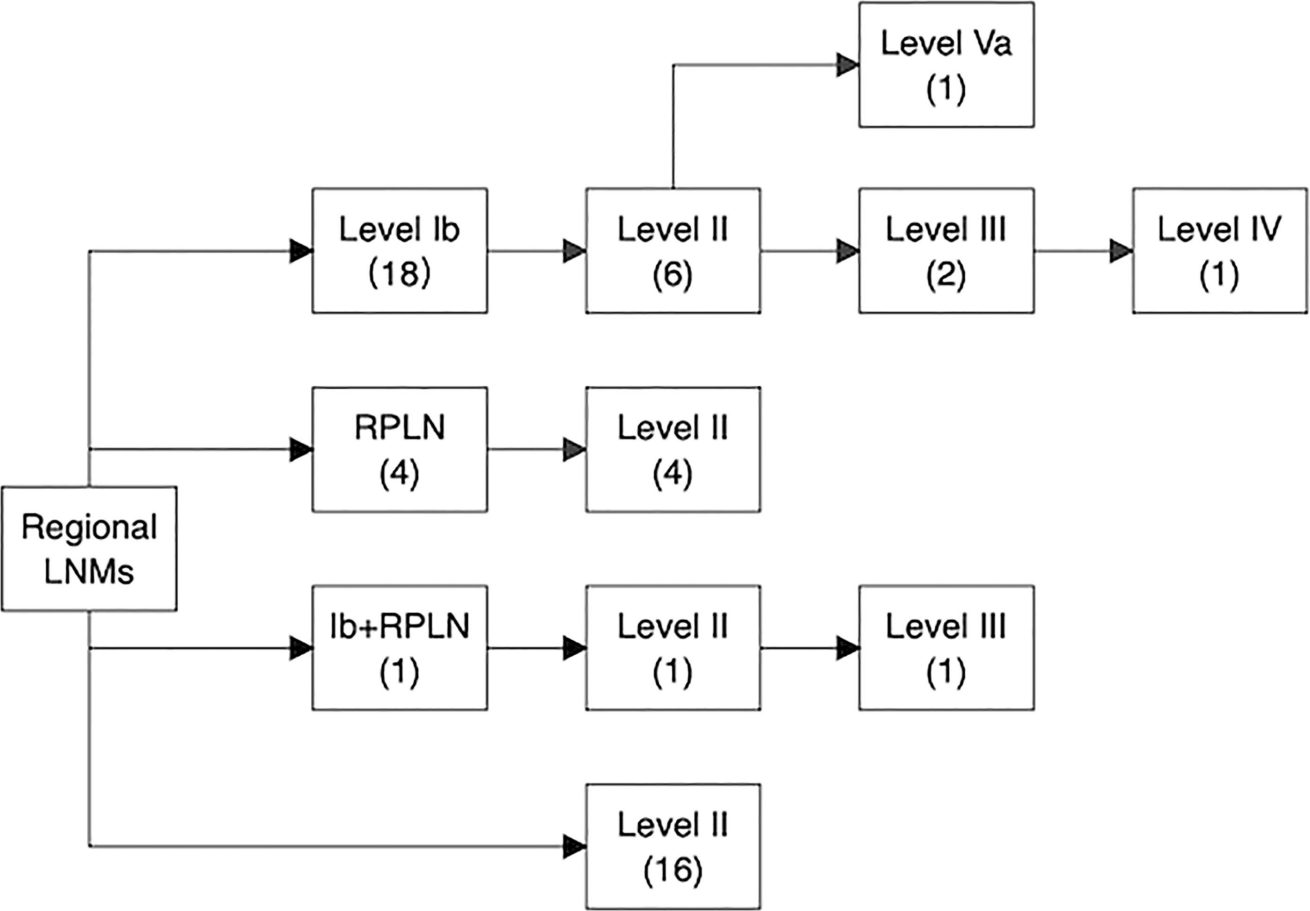
Pathways of ipsilateral lymph node spread in SNSCC. The number in the bracket represents the number of patients who had nodal involvement in specific lymph node levels.

Of these 53 patients, 73.6% had ipsilateral LNM, and 26.4% had bilateral LNM, while isolated contralateral LNM was not observed. The most frequently involved sites were level II LNs (41/255, 16.1%), followed by level Ib LNs (26/255, 10.2%) and RPLNs (12/255, 4.7%). Middle and lower jugular LN involvement was rare (level III LNs: 3.2%, level Iva LNs: 1.4%). In addition, metastatic LNs at level Va were observed in 4 (1.6%) patients, and only 2 (0.8%) patients had metastatic level VIII (preauricular) LNs. We further analyzed the spread of ipsilateral clinically metastatic lymph nodes (**Figure 5**) and found that no patient presented with skip metastasis.

#### LNM Rate and Spread Pattern of LNM During Follow-Up

Of all 255 patients, 19 (7.5%) patients (with involvement of the nasal cavity, 7/76, 9.2%; with involvement of the maxillary sinus, 9/148, 6%; with involvement of the ethmoid sinus, 3/30, 10%) experienced regional recurrence (RR), and 84% (16/19) developed RR during the first two years of follow-up. Detailed information on the 19 patients with nodal relapse is shown in **Table 4**. Isolated RR was present in 5 (2%) patients, and 4 of them successfully underwent salvage surgery.

**Table 4:** Details of 19 patients with regional recurrence.

Pt. no	Primary tumor site	Stage	Tx	NeckTx	ENI target volume	Failure event	Neck failure location	Neck salvage Tx	Neck salvage results	Status following salvage therapy
1	Maxillary sinus	T2N1	S+RT	S^#^		N	contra: II	S	CR	Died of renal failure
2	Nasal cavity	T2N2c	RT	RT	ipsi: Ib, II, III, IV, V; contra: Ib, II, III, IV, V.	N	ipsi: II, III, IV, V; contra: II, III, IV, V, RPLN.	S	CR	Died from accident
3	Nasal cavity	T4aN3b	RT	RT	ipsi: Ib, II, III, IV, V; contra: Ib, II, III, IV, V.	N	ipsi: Ib.	S	CR	Alive
4	Maxillary sinus	T3N0	S+RT	ENI	ipsi: Ib, II, III; contra: II.	T+N	ipsi: Ib, II, III; contra: Ib, II, III.	S	CR	Alive
5	Nasal cavity	T4aN0	S+RT	ENI	ipsi: RPLN; contra: RPLN.	T→N→M	ipsi: Ib, II; contra: II.	S	CR	Died of cancer
6	Ethmoid sinus	T4bN0	RT	ENI	ipsi: II, III, IV; contra: II, III, IV.	T+N+M	ipsi: Ib; contra: Ib.	Chemotherapy		Died of cancer
7	Maxillary sinus	T4aN0	S+RT	ENI	ipsi: II; contra: II.	T+N+M	ipsi: II.	Chemotherapy	SD	Died of cancer
8	Maxillary sinus	T4bN0	S+RT	ENI	ipsi: II;	T+N+M	ipsi: Ib, VIII.	_		Died of cancer
9	Maxillary sinus	T4aN0	RT+S	ENI	ipsi: Ib, II, III;	N	ipsi: II.	S	CR	
10	Maxillary sinus	T4aN0	RT+S	ENI	ipsi: Ib, II, III, RPLN; contra: Ib, II, III, RPLN.	N	ipsi: IVa.	_		Died of intercurrent diseases
11	Ethmoid sinus	T4bN0	RT	ENI	ipsi: II, III; contra: II, III.	N→2thN→M	ipsi: II.	S	CR	Died of cancer
12	Ethmoid sinus	T4bN0	S+RT	ENI	ipsi: Ib, II, III, IV; contra: Ib, II, III, IV.	N→T→M	ipsi: II.	S+RT	CR	Died of cancer
13	Nasal cavity	T4bN0	RT	ENI	ipsi: Ib, II.	N+M	ipsi: II; contra: II.	_		Died of cancer
14	Maxillary sinus	T4bN0	S+RT	OBS		T+N	ipsi: Ib.	_		Died of cancer
15	Maxillary sinus	T3N0	S+RT	OBS		T+N	ipsi: II.	S	CR	Died of cancer
16	Maxillary sinus	T4bN0	RT+S	OBS		T+N→T	contra: II.	S	CR	Died of cancer
17	Nasal cavity	T4bN0	S+RT	OBS		T→N	ipsi: IVb; contra: Ib.	RT	PD	Died of intercurrent diseases
18	Nasal cavity	T4aN0	S+RT	OBS		T→N	ipsi: II, VIII.	_		Died of cancer
19	Nasal cavity	T4aN0	S+RT	OBS		T→N+M	ipsi: II, III; contra: II, III.	Chemotherapy	PD	Died of cancer

Pt, Patients; Tx, Treatment; S, Surgery; RT, Radiotherapy; ENI, Elective Neck Irradiation; OBS, observation; T, Local failure; N, Nodal failure; M, Distant metastasis; ipsi, ipsilateral; contra, contralateral; CR, complete response; PD, progressive disease.

^#^No.1 patient underwent the ipsilateral neck dissection.

Of the patients with delayed appearance of metastatic LNs, 11 patients had the metastases develop in the ipsilateral neck, 7 had the metastases develop in the bilateral neck, and 1 patient developed nodal failure in the contralateral neck alone. Level II LNs were the most involved lymphatic site, with 18 patients showing level II LN involvement, followed by level Ib LNs (7 patients), level III LNs (3 patients), level IV LNs (3 patients), level VIII LNs (2 patients), level Va LNs (1 patient), and RPLNs (1 patient).

### Risk Factors for LNM

Multivariate logistic analysis (**Table 5**) revealed that nasopharyngeal invasion was associated with a higher rate of initial LNM (OR = 3.43, 95%CI: 1.435-8.196, p = 0.006).

**Table 5:** Multivariate logistic analysis of risk factors for initial lymph node metastasis.

Variables	Initial LNMs(all patients)	%	OR	95%CI		p	Delayed LNMs(N0 patients)	%	HR	95%CI		p
Age												
≤50	21/100	21.00%	1				8/79	10.10%	1			
>50	32/155	20.60%	0.734	0.356	1.511	0.401	8/123	6.50%	0.284	0.068	1.185	0.084
Sex												
Male	43/190	22.60%	1				12/147	8.20%	1			
Female	10/65	15.40%	0.617	0.255	1.493	0.284	4/55	7.30%	0.562	0.151	2.098	0.392
Primary site						0.196						0.922
Nasal cavity	20/76	26.30%	1				5/56	8.90%	1			
Maxillary sinus	29/149	19.50%	0.505	0.216	1.178	0.114	8/120	6.70%	1.349	0.171	10.632	0.776
Ethmoid sinus	4/30	13.30%	0.418	0.216	1.178	0.244	3/26	11.50%	1.213	0.235	6.261	0.818
T stage						0.670						0.985
T1	0/5	0.00%	0	0	–	0.999	0/5	0.00%	0	0	–	0.992
T2	4/15	26.70%	2.623	0.574	11.98	0.213	0/11	0.00%	0	0	–	0.988
T3	9/56	16.10%	1.304	0.436	3.904	0.635	2/47	4.30%	0.669	0.089	5.036	0.696
T4	40/179	22.30%	1				14/139	10.10%	1			
Orbit invasion												
Yes	35/170	20.60%	0.705	0.275	1.807	0.466	14/135	10.40%	4.185	0.69	25.365	0.119
No	18/85	21.20%	1				2/67	3.00%	1			
Pterygopalatine fossa invasion												
Yes	29/97	29.90%	1.569	0.608	4.047	0.352	2/68	2.90%	0.044	0.004	0.533	0.014
No	24/158	15.20%	1				14/134	10.40%	1			
Infratemporal fossa invasion												
Yes	22/93	23.70%	0.805	0.297	2.18	0.669	5/71	7.00%	1.526	0.199	11.705	0.684
No	31/162	19.10%	1				11/131	8.40%	1			
Dura invasion												
Yes	11/32	34.40%	2.831	0.934	8.576	0.066	3/21	14.30%	2.407	0.455	12.722	0.301
No	42/223	18.80%	1				13/181	7.20%	1			
Nasopharynx invasion												
Yes	22/48	45.80%	3.43	1.435	8.196	0.006	3/26	11.5%	11.736	1.352	101.857	0.026
No	31/207	15.00%	1				13/176	7.40%	1			
Hard palate invasion												
Yes	24/75	32.00%	0.748	0.231	2.415	0.627	4/51	7.80%	0.884	0.082	9.517	0.919
No	29/180	16.10%	1				12/151	7.90%	1	0.566	12.701	0.214
Soft palate invasion												
Yes	6/12	50.00%	1.879	0.435	8.108	0.398	0/6	0.00%	1			
No	47/243	19.30%	1				16/196	8.20%	0	0	.	0.994
Oral cavity invasion												
Yes	28/89	31.50%	2.248	0.435	8.108	0.161	5/61	8.10%	2.097	0.178	24.627	0.556
No	25/165	15.20%	1				11/140	7.90%	1	0.151	2.098	0.392
Facial soft tissue invasion												0.922
Yes	23/74	31.10%	2.106	0.932	4.758	0.073	6/51	11.80%	2.682	0.566	12.701	0.214
No	30/181	16.60%	1				10/151	6.60%	1	0.171	10.632	0.776
Treatment modality												
S+RT							10/84	11.90%	1			
RT+S							3/61	4.90%	0.239	0.048	1.184	0.079
RT							3/45	6.70%	0.517	0.105	2.545	0.417
S							0/12	0%	0	0	–	0.986
Chemotherapy												
Yes							4/50	7.40%	0.964	0.310	2.996	0.949
No							12/136	8.10%	1			
Neck Treatment												
ENI							10/147	6.80%	0.169	0.041	0.690	0.013
OBS							6/55	10.90%	1			

For delayed lymph node recurrence during follow-up, in addition to ENI, predictors also included nasopharyngeal involvement (HR = 11.736, 95%CI: 1.352-101.857, p = 0.026); however, although it reached the significance level, the wide confidence intervals may influence the statistical power. Pterygopalatine fossa involvement was associated with a lower rate of RR (HR = 0.033, 95%CI: 0.004-0.533, p = 0.014).

## Discussion

In the present study, we confirmed that regional LNM was a negative prognostic factor for survival in SNSCC (5y-OS, N0: 55.7%; N+ of N-relapse: 38.5%). In our cohort, prophylactic neck irradiation was associated with a lower rate of RR after PSM. We also found that the rate of initial LNM was 20.8%, and the highest rate presented in nasal cavity tumors was 26.3%, while the delayed nodal recurrence rate was 7.5%. In addition, lymphatic spreading followed orderly patterns. Last, nasopharynx and pterygopalatine fossa involvement were independent factors for predicting RR.

Despite recent advances in therapeutic technology, the treatment of SNSCC remains a challenge. The inferior prognosis caused by regional lymph node issues is worth noting. In this series, the 5-year OS was 55.7% in patients with an initial N0 neck vs. 38.5% in those with LNM at diagnosis or after treatment, which are similar to the findings in previous studies, in which the 5-year OS and DSS were reduced by 10~40% for patients with N+ compared with N0 disease (5–7).

ENI resulted in a lower regional failure rate than OBS, as revealed by the PSM analysis and multivariate Cox regression analysis. The fact that a majority of (72.8%) patients underwent ENI may explain why the rate of RR was slightly lower in our findings than in others. Abu-Ghanem et al. (13) summarized publications since the 1900s and found that nodal recurrence was detected in 0~4.3% of the patients in the ENI group and in 9.1%~33% of patients in the OBS group. The researchers indicated that ENI could significantly reduce the nodal recurrence rate for patients with maxillary sinus SCC. In nasal cavity SCC, Ahn et al. (18) reported a nodal recurrence rate of 18.8% for N0 patients with no ENI.

Similarly, another meta-analysis found an 18.1% RR rate for nasal cavity tumors with or without ENI. The study also suggested that ENI is an effective method for reducing RR (19). The same conclusion was also drawn by Galloni et al. (20). Moreover, Jiang et al. and Paulino et al. reported high RR rates of 33% and 28.9%, respectively, for N0 patients, and a lack of ENI was associated with significantly worse survival (10, 11). As a result, MD Anderson Cancer Center changed its guidance to include irradiation of the neck in T2-4 maxillary SCC.

The researchers indicated that most patients with failure in the neck have simultaneous or preceding local failure, and the rate of isolated RR was low at 0%-16.7% (21). We hypothesized that the local lesion is potentially the source of metastatic dissemination to lymph nodes. Besides, due to the rarity and high salvageability of isolated RR, some investigators oppose routine ENI (15, 22). Regarding the especially high LNM rates found by Paulino et al. (11), in that study, the isolated neck failure rate was only 10.5%. This low rate of isolated nodal failure was also found by Mirghani et al (23), ranging from 2.8% to 13% (23). Moreover, in these isolated RR cases, salvage treatment results in good oncologic outcomes. Cantù et al. (6) reported that 97% (28/30) of patients were successfully salvaged. Similarly, both Dirix (24) and Porceddu (25) reported a high salvageability of over 50%. Notably, in a multi-institutional study (15), among 5 patients with isolated LNM, 3 patients were successfully salvaged, and 2 failed because of RPLN metastasis. Thus, the prophylactic irradiation of RPLNs deserves consideration because salvage surgery is difficult. In our results, the rate of isolated RR was only 2%, but the effectiveness of ENI indicated that the rate of isolated RR might be unequal to that of occult LNM at diagnosis.

The incidence of lymph node metastasis (LNM) at presentation or during follow-up varies widely from 3% to 33%. The initial incidence of LNM was 20.8% in the current study. According to the primary site, the rate of initial LNM was 26.3% in the nasal cavity, 19.5% in the maxillary sinus and 13.3% in the ethmoid sinus. Ahn et al. revealed rates of 7.9% and 15.2% for nasal cavity and maxillary sinus nodal involvement, respectively, in 2888 patients (18). A meta-analysis of nasal cavity SCC identified a rate of initial LNM of 0~27% (below the 10% rate in most of the enrolled articles) (19), and another study showed a rate of 3~20.6% (10%~20% in most of the enrolled articles) in maxillary sinus SCC (13). Similar results depending on site were also found in evaluations of the SEER (26) and NCDB (27) databases. Cantù et al. (6) reported that 305 ethmoid sinus tumors had 1.6% rate of nodal metastasis at presentation. Contrary to their findings, we found that nasal cavity tumors had the highest rate of initial LNM, and the rate of LNM in ethmoid sinus tumors was higher than that in others’ results. One reason for this finding is that there were fewer early-stage tumors in our study than other studies, and the tumor staging was the most important factor for dissemination including lymph nodes and distant organs. Due to the medical diversion system, as a tertiary hospital, the proportion of advanced-stage patients treated in our hospital has increased in recent years (**eTable 2**). This might explain why there were more advanced-stage patients in our hospital than in others. In the present study, 6.6% of patients had T1 disease; while in Dutta’s study, Becker’s study and Cantù’s study, 44.9%, 38.5%, and 24% of patients had T1 disease (6, 26, 28). While the rate of LNM at diagnosis was higher in our study, the incidence of RR (7.5%) was lower than that in most of the existing studies. We consider that the low RR rate of cervical lymph nodes is due to the fact that 72.8% of patients have received ENI.

If ENI is to be delivered, there is no consensus about the optimal neck irradiation volume or dose for ENI. The nasal cavity and paranasal sinuses are thought to be areas with two main pathways of lymphatic drainage: the anterior route runs around the facial artery vessels, draining into the submandibular nodes (level I); and the posterior route runs to the upper jugular nodes (level 2) through retropharyngeal or parapharyngeal nodes (29). Recently, Fernández et al. performed lymphoscintigraphy during sentinel lymph node biopsy for patients with sinonasal tumors and found that levels I and II most commonly contained the sentinel node (14).

The distribution of LNM in this study was consistent with previous research. The most common levels involved were levels II and Ib of the ipsilateral neck. Level IV or V nodal involvement was observed in a minority of patients with level II and III nodal metastasis, which may be related to the intrinsic aggressiveness of the individual disease or the advanced stage of the primary tumor (18). Notably, the incidence of recurrence in RPLNs was the lowest of all the regions after treatment, though the incidence of RPLN recurrence ranked third at presentation. It is possible that the retropharyngeal space received radiation doses as a CTV or outside the CTV enough to lead the occult metastasis to cause death. Guan et al (30) found that 18.6% (11/59) of patients had RPLN involvement at diagnosis, but only 1 patient developed RPLN recurrence during follow-up. Dosimetric analysis showed that the median CTV dose delivered to the retropharyngeal space was 43.3 Gy. Therefore, the researchers suggested that only ipsilateral levels Ib and II be prophylactically irradiated. However, Gangl et al (31) reported that the rate of initial RPLN involvement was 45.5% (10/23) in SNSCC, and it was a prognostic factor for OS and locoregional control. Regretfully, the incidence of RPLN recurrence after treatment was not provided. Thus, whether RPLNs are routinely included in prophylactic irradiation fields and the optimal dose need to be assessed in future studies.

In our study, the multivariate analysis showed that nasopharyngeal invasion was a risk factor for initial or delayed LNM. Similarly, Homma et al (15) reported that involvement of the nasopharynx was correlated with LNM, and involvement of the hard palate was also identified. These two areas are known to be rich in lymphatic networks that can lead to LNM development. Regarding the pterygopalatine fossa, the sample size may be a reason for the decreased RR rate.

We acknowledge that our analysis had limitations inherent to retrospective studies, such as the small number of RRs may have limited the statistical power to identify some other associations, although our study included larger sample sizes of Asian populations with significant long-term follow-up outcomes. In addition, some patients received preoperative RT in this article, which is inconsistent with the practice of surgery combined with postoperative RT adopted by most international institutions, but it does not violate the multimodal therapy recommended in advanced disease by the NCCN guidelines. As the largest cancer treatment type in Asia, the preoperative RT strategy has been successfully utilized in head and neck squamous cell carcinoma for decades (32, 33). The results of clinical practice have shown that planned preoperative RT can improve the orbital retention rate without affecting the survival outcomes (34, 35). Moreover, in this study, the proportion of patients receiving preoperative and postoperative RT was similar in the OBS group and ENI group.

In conclusion, patients who developed lymph node metastasis at diagnosis or during follow-up had poorer survival. The rate of LNM was consistent with previous studies, and lymphatic spreading in SNSCC followed predictable patterns. Prophylactic neck irradiation could effectively reduce the rate of RR in patients with SNSCC.

## Data Availability Statement

The raw data of this article will be made available by the corresponding author upon appropriate request.

## Ethics Statement

This observational study was carried out following the declaration of Helsinki and approved with exemption from informed consent by the Independent Ethics Committee of Cancer Hospital, Chinese Academy of Medical Sciences.

## Author Contributions

QL contributed conception and design of the study; QL organized the database; QL performed the statistical analysis; QL wrote the first draft of the manuscript; QL wrote sections of the manuscript. All authors contributed to the article and approved the submitted version.

## Conflict of Interest

The authors declare that the research was conducted in the absence of any commercial or financial relationships that could be construed as a potential conflict of interest.

## Publisher’s Note

All claims expressed in this article are solely those of the authors and do not necessarily represent those of their affiliated organizations, or those of the publisher, the editors and the reviewers. Any product that may be evaluated in this article, or claim that may be made by its manufacturer, is not guaranteed or endorsed by the publisher.

## Supplementary Material

Supplementary Figure 1 Kaplan-Meier estimates of the cumulative incidence of regional recurrence **(A)** and overall survival **(B)** in different RT technologies. Among 147 patients who received ENI, 94 patients were treated with IMRT, and 53 patients were treated with 3D-CRT. We depicted the KM survival curve of cumulative incidence of regional recurrence and compared IMRT and 3D-CRT using the log-rank test. There was no significant difference between IMRT and 3D-CRT on RR.

Supplementary Figure 2 Kaplan-Meier estimates of the cumulative incidence of regional recurrence **(A)** and overall survival **(B)** in different diagnosis time periods. **(A)** Regional Recurrence in different diagnosis time periods. **(B)** Overall Survival in different diagnosis time periods.

Supplementary Table 1 Univariate and multivariate analysis of risk factors associated with overall survival in 202 patients with N0 neck. S, Surgery; RT, Radiotherapy.

Supplementary Table 2 The proportion of tumor stage and neck treatment in different diagnosis time.
