# Supplementary figures and images for: Lymph Node Metastasis Spread Patterns and the Effectiveness of Prophylactic Neck Irradiation in Sinonasal Squamous Cell Carcinoma (SNSCC)
Source: Front Oncol. 2022 May 30;12:793351. doi: 10.3389/fonc.2022.793351 (PMC9190260; doi:10.3389/fonc.2022.793351)

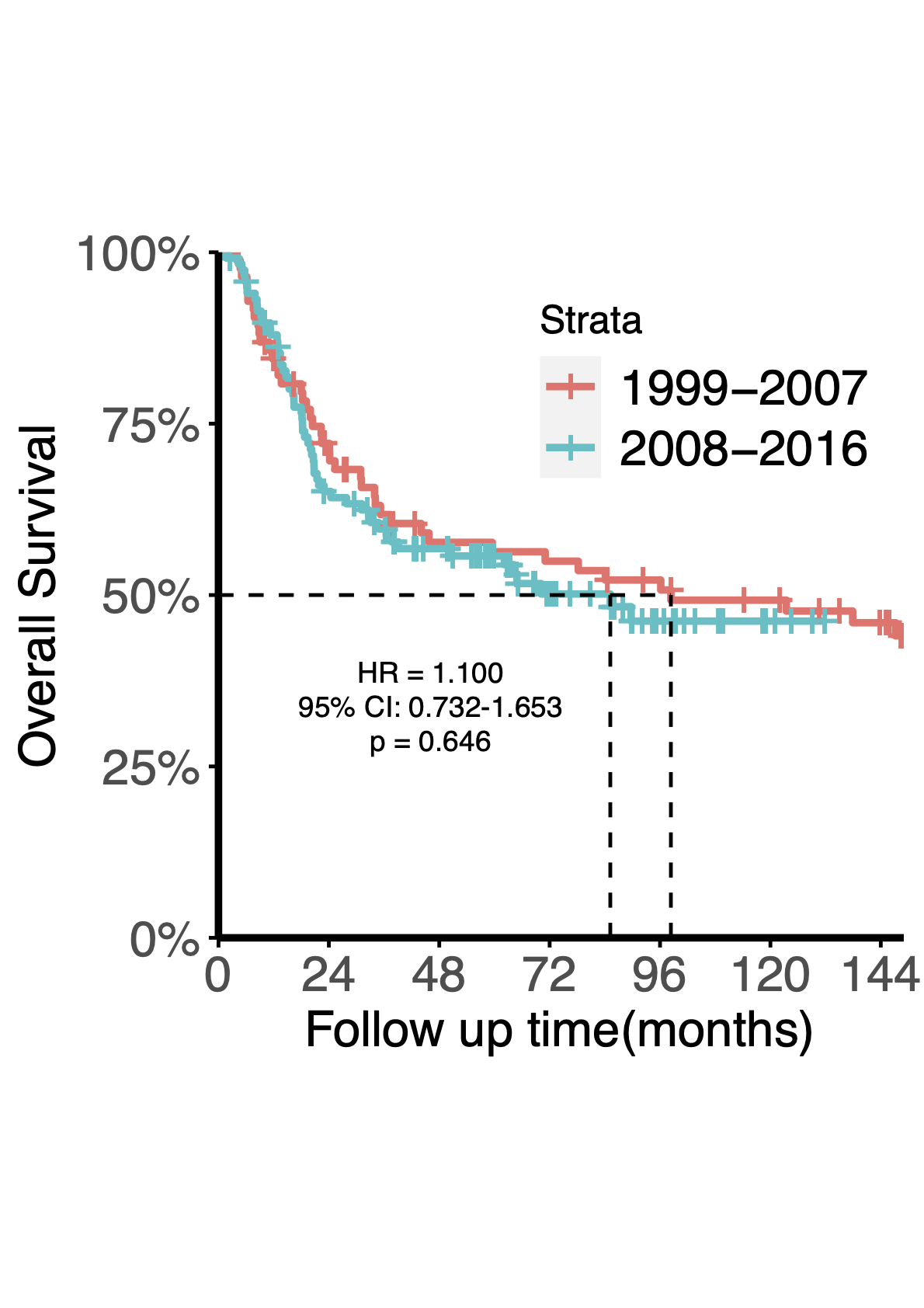

Supplement: Supplementary Figure 1 — Kaplan-Meier estimates of the cumulative incidence of regional recurrence (A) and overall survival (B) in different RT technologies. Among 147 patients who received ENI, 94 patients were treated with IMRT, and 53 patients were treated with 3D-CRT. We depicted the KM survival curve of cumulative incidence of regional recurrence and compared IMRT and 3D-CRT using the log-rank test. There was no significant difference between IMRT and 3D-CRT on RR. [file Image_1.tiff]

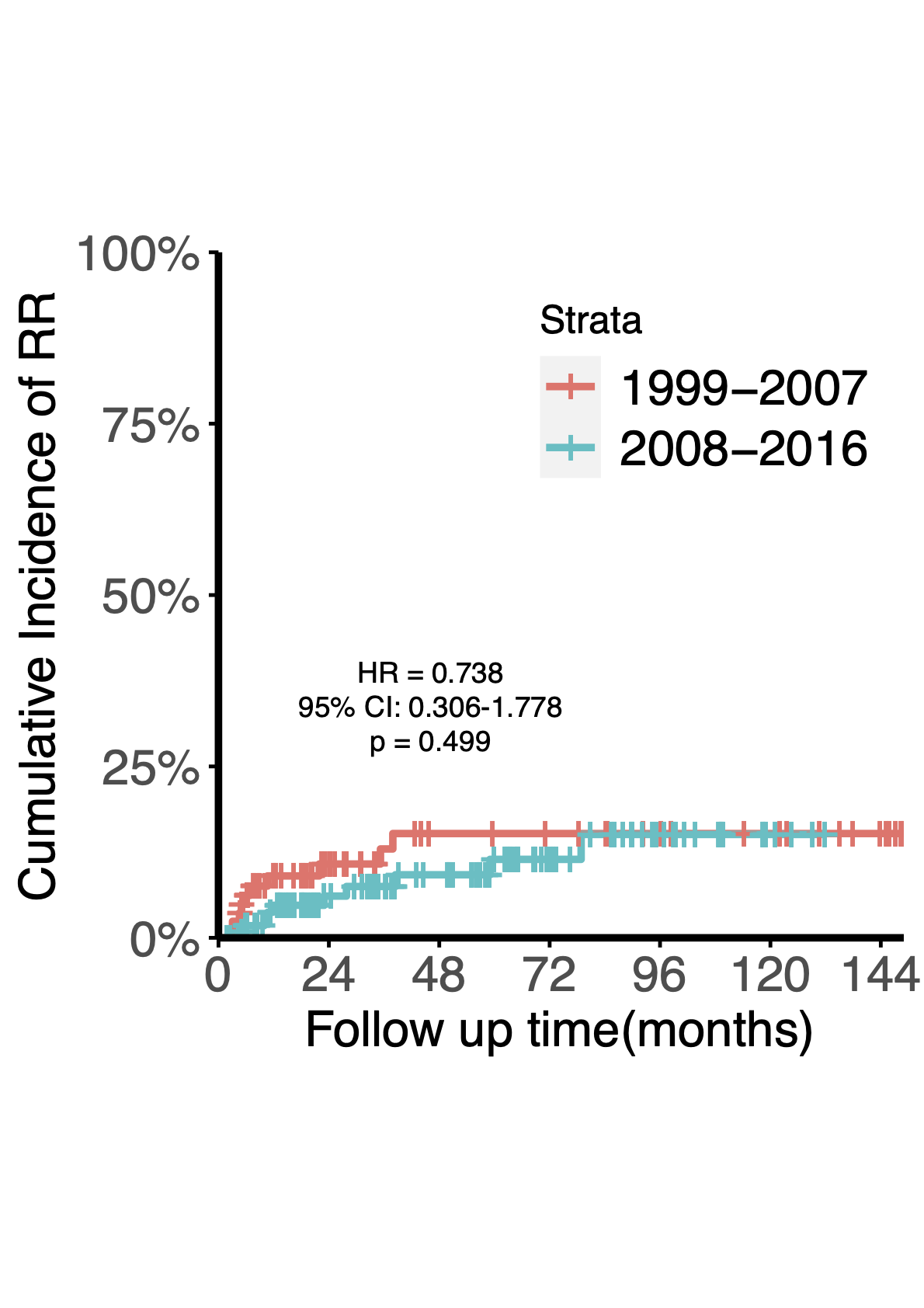

Supplement: Supplementary Figure 2 — Kaplan-Meier estimates of the cumulative incidence of regional recurrence (A) and overall survival (B) in different diagnosis time periods. (A) Regional Recurrence in different diagnosis time periods. (B) Overall Survival in different diagnosis time periods. [file Image_2.tiff]

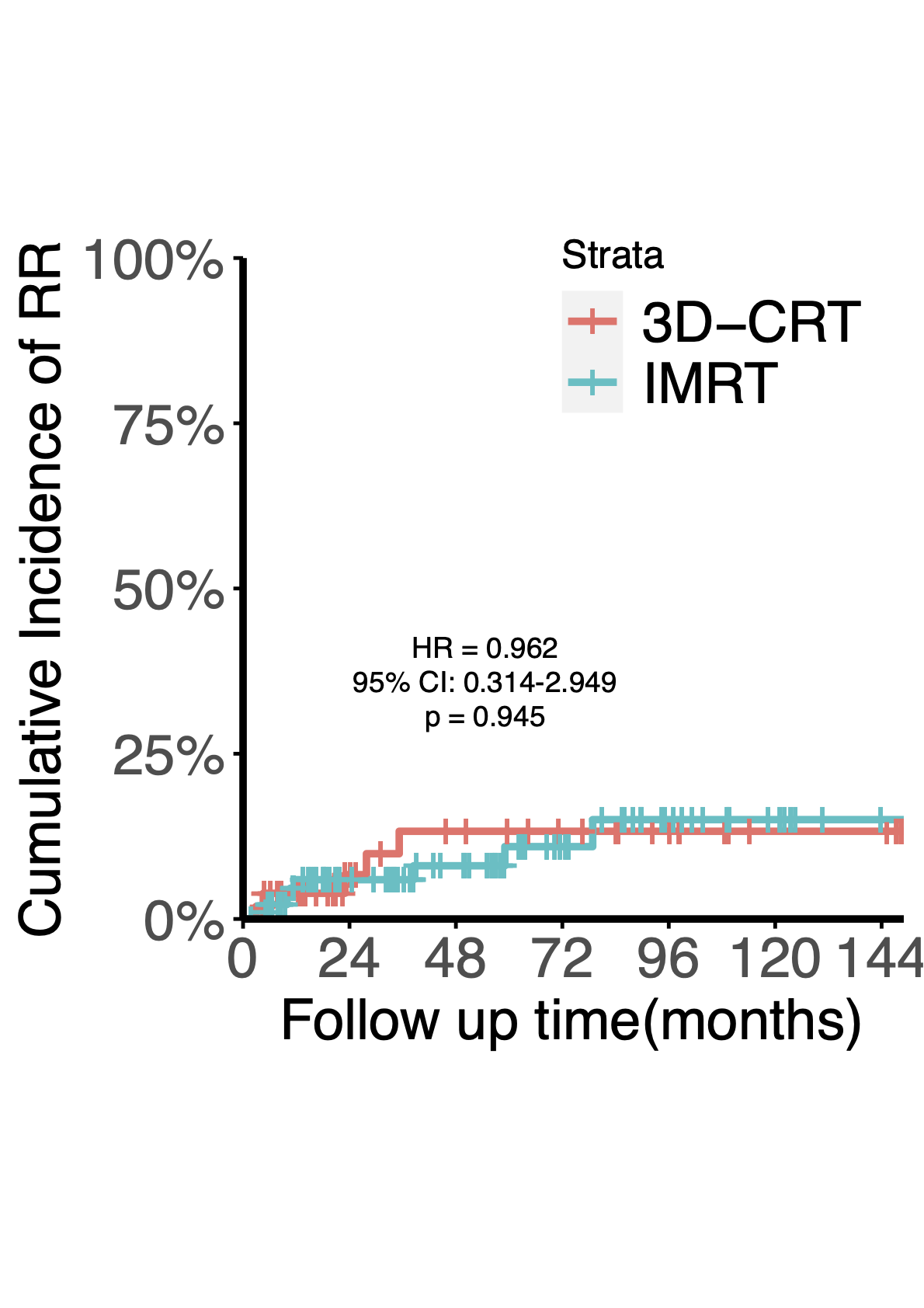

Supplement: Supplementary file 3 [file Image_3.tiff]
