# Supplementary material for: Lymph Node Metastasis Spread Patterns and the Effectiveness of Prophylactic Neck Irradiation in Sinonasal Squamous Cell Carcinoma (SNSCC)
Source: Front Oncol. 2022 May 30;12:793351. doi: 10.3389/fonc.2022.793351 (PMC9190260; doi:10.3389/fonc.2022.793351)
Supplement: Supplementary Table 1 — Univariate and multivariate analysis of risk factors associated with overall survival in 202 patients with N0 neck. S, Surgery; RT, Radiotherapy. [file Table_1.docx]

**Supplement**

**eTable 1. Univariate and multivariate analysis of risk factors associated with overall survival in 202 patients with N0 neck**

|  | |  | | Univariate | | |  | | |  | | Multivariate | | | | |  |
| --- | --- | --- | --- | --- | --- | --- | --- | --- | --- | --- | --- | --- | --- | --- | --- | --- | --- |
| Variables | n (%) | | HR | | 95%CI | | | p | | | HR | | | 95%CI | p | | |
| Age |  | |  | |  | | |  | | |  | | |  |  | | |
| ≤50 | 79 (39.1) | | 1 | |  | | |  | | |  | | |  |  | | |
| >50 | 123 (60.9) | | 1.05 | | 0.718-1.55 | | | 0.787 | | |  | | |  |  | | |
| Sex |  | |  | |  | | |  | | |  | | |  |  | | |
| Male | 147 (72.8) | | 1 | |  | | |  | | |  | | |  |  | | |
| Female | 55 (27.2) | | 0.805 | | 0.524-1.24 | | | 0.323 | | |  | | |  |  | | |
| Chemotherapy | |  | |  | |  | | |  | | | |  | | |  | |
| No | 148 (73.3) | |  | |  | | |  | | |  | | |  |  | | |
| Yes | 54 (26.7) | | 1.49 | | 0.996-2.23 | | | 0.052 | | |  | | |  |  | | |
| Neck Treatment | |  | |  | |  | | |  | | | |  | | |  | |
| OBS | 55 (27.2) | | 1 | |  | | |  | | |  | | |  |  | | |
| ENI | 147 (72.8) | | 1.07 | | 0.697-1.65 | | | 0.749 | | |  | | |  |  | | |
| Primary site | |  | |  | |  | | |  | | | |  | | |  | |
| Nasal cavity | 56 (27.7) | | 1 | |  | | |  | | |  | | |  |  | | |
| Maxillary sinus | 120 (59.4) | | 0.928 | | 0.635-1.35 | | | 0.697 | | | 1.008 | | | 0.25-0.033 | 0.973 | | |
| Ethmoid sinus | 26 (12.9) | | 1.98 | | 1.22-3.19 | | | 0.005 | | | 1.193 | | | 0.321-0.552 | 0.581 | | |
| T stage |  | |  | |  | | |  | | |  | | |  |  | | |
| T1 | 5 (2.5) | | 1 | |  | | |  | | |  | | |  |  | | |
| T2 | 11 (5.4) | | 0.543 | | 0.2-1.48 | | | 0.232 | | | 1.249 | | | 0.893-0.249 | 0.804 | | |
| T3 | 47 (23.3) | | 0.353 | | 0.201-0.619 | | | ＜0.001 | | | 0.898 | | | 0.786-0.136 | 0.892 | | |
| T4 | 139 (68.8) | | 2.94 | | 1.81-4.79 | | | ＜0.001 | | | 2.818 | | | 0.773-1.34 | 0.18 | | |
| Treatment modality | |  | |  | |  | | |  | | | |  | | |  | |
| S+RT | 84 (41.6) | | 1 | |  | | |  | | |  | | |  |  | | |
| RT+S | 61 (30.2) | | 0.729 | | 0.478-1.11 | | | 0.144 | | | 0.986 | | | 0.25-0.054 | 0.957 | | |
| RT | 45 (22.3) | | 2.81 | | 1.88-4.22 | | | ＜0.001 | | | 2.671 | | | 0.254-3.864 | ＜0.001 | | |
| S | 12 (5.9) | | 0.883 | | 0.41-1.9 | | | 0.751 | | | 1.777 | | | 0.435-1.32 | 0.187 | | |

Abbreviation: S, Surgery; RT, Radiotherapy.
