# Supplementary material for: Lymph Node Metastasis Spread Patterns and the Effectiveness of Prophylactic Neck Irradiation in Sinonasal Squamous Cell Carcinoma (SNSCC)
Source: Front Oncol. 2022 May 30;12:793351. doi: 10.3389/fonc.2022.793351 (PMC9190260; doi:10.3389/fonc.2022.793351)
Supplement: Supplementary Table 2 — The proportion of tumor stage and neck treatment in different diagnosis time. [file Table_2.docx]

**eTable 2.** **The proportion of tumor stage and neck treatment in different diagnosis time**

|  | 1999-2007  n = 84 (%) | 2008-2016  n = 118 (%) | p |
| --- | --- | --- | --- |
| T stage |  |  | 0.013 |
| T1 | 4 (4.8) | 1 (0.8) |  |
| T2 | 7 (8.3) | 4 (3.4) |  |
| T3 | 25 (29.8) | 22 (18.6) |  |
| T4 | 48 (57.1) | 91 (77.1) |  |
| Neck Treatment |  |  | ＜0.001 |
| OBS | 34 (40.5) | 21 (17.8) |  |
| ENI | 50 (59.5) | 97 (82.2) |  |
